# Deoxycholate promotes survival of breast cancer cells by reducing the level of pro-apoptotic ceramide

**DOI:** 10.1186/bcr2211

**Published:** 2008-12-16

**Authors:** Kannan Krishnamurthy, Guanghu Wang, Dmitriy Rokhfeld, Erhard Bieberich

**Affiliations:** 1 Institute of Molecular Medicine and Genetics, School of Medicine, Medical College of Georgia, 15th Street, Augusta, GA 30912, USA; 2 Student Research and Training (STAR) Program, School of Graduate Studies, Medical College of Georgia, 15th Street, Augusta, GA 30912, USA

## Abstract

**Introduction:**

At physiologic concentration in serum, the bile acid sodium deoxycholate (DC) induces survival and migration of breast cancer cells. Here we provide evidence of a novel mechanism by which DC reduces apoptosis that is induced by the sphingolipid ceramide in breast cancer cells.

**Methods:**

Murine mammacarcinoma 4T1 cells were used *in vitro *to determine apoptosis and alteration of sphingolipid metabolism by DC, and *in vivo *to quantify the effect of DC on metastasis.

**Results:**

We found that DC increased the number of intestinal metastases generated from 4T1 cell tumors grafted into the fat pad. The metastatic nodes contained slowly dividing cancer cells in immediate vicinity of newly formed blood vessels. These cells were positive for CD44, a marker that has been suggested to be expressed on breast cancer stem cells. In culture, a subpopulation (3 ± 1%) of slowly dividing, CD44^+ ^cells gave rise to rapidly dividing, CD44^- ^cells. DC promoted survival of CD44^+ ^cells, which was concurrent with reduced levels of activated caspase 3 and ceramide, a sphingolipid inducing apoptosis in 4T1 cells. Z-guggulsterone, an antagonist of the farnesoid-X-receptor, obliterated this anti-apoptotic effect, indicating that DC increased cell survival via farnesoid-X-receptor. DC also increased the gene expression of the vascular endothelial growth factor receptor 2 (Flk-1), suggesting that DC enhanced the initial growth of secondary tumors adjacent to blood vessels. The Flk-1 antagonist SU5416 obliterated the reduction of ceramide and apoptosis by DC, indicating that enhanced cell survival is due to Flk-1-induced reduction in ceramide.

**Conclusions:**

Our findings show, for the first time, that DC is a natural tumor promoter by elevating Flk-1 and decreasing ceramide-mediated apoptosis of breast cancer progenitor cells. Reducing the level or effect of serum DC and elevating ceramide in breast cancer progenitor cells by treatment with Z-guggulsterone and/or vascular endothelial growth factor receptor 2/Flk-1 antagonists may thus be a promising strategy to reduce breast cancer metastasis.

## Introduction

Breast cancer metastasis is a multi-step process that starts with evasion of cancer cells from the primary tumor, migration through the blood stream and lymphatic ducts, and homing into critical target tissues, creating a life-threatening disease [1]. In all of these steps, cancer cells are exposed to growth factors and chemokines in the serum, vastly enhancing their potential to proliferate and migrate. Most recently, our laboratory has shown that one of these factors is the bile acid sodium deoxycholate (DC) [1]. DC is synthesized by bacteria in the intestine and, like other bile acids, it facilitates dietary fat processing. In blood, the normal serum concentration of DC is about 5 to 10 μmol/l, but increased levels of more than 50 μmol/l have been determined in breast cyst fluid [2,3]. Historically, DC was among the first bile acids found to cause cancer, and it has been shown to promote colon carcinogenesis [4,5]. Despite this evidence that DC is a physiological tumor promoter, little is known about its effect on various types of cancer cells.

Previously, we showed that 10 μmol/l DC enhances survival and migration of human breast cancer MDA-MB-231 cells by fivefold and 60%, respectively [1]. This anti-apoptotic and chemokinetic effect was unexpected because numerous other studies found that DC induced apoptosis in cancer cells [6-9]. The pro-apoptotic effect, however, relied on incubation with up to 50-fold higher concentrations of DC than used in our study. Our laboratory was also the first to show that the bile acid receptor farnesoid X receptor (FXR) is expressed in breast cancer cells, that DC induced translocation of FXR into the nucleus, and that this increased the protein levels of urokinase like plasminogen activator (uPA) and its receptor [1]. In particular, expression of uPA and uPA receptor are common to metastatic cancers and help cancer cells to invade solid tissues. The elevated gene expression of FXR as a tumor marker in breast cancer has been confirmed by other groups [10-12]. It is not known, however, whether DC will also promote breast cancer cell migration and metastasis *in vivo*.

In this study we used a well established orthotopic, syngeneic, and metastatic murine 4T1 breast cancer model in Balb/c mice [13-18]. The majority of metastatic tumor nodes were observed in the intestine and liver, two tissues with high concentrations of DC. DC promoted survival and growth of slowly dividing cancer cells that could self-renew and gave rise to rapidly dividing breast cancer cells. The slowly dividing cells expressed high levels of the vascular endothelial growth factor (VEGF) receptor 2 (Flk-1) and the hyaluronic acid receptor CD44 [19-21]. These data suggested that DC promoted the proliferation of breast cancer cells, which formed metastases at newly formed blood vessels. Although the expression of CD44 has been attributed to a cancer stem cell-like phenotype and found to increase the invasive properties of breast cancer cells, its role and prevalence in metastasis have been controversial [19,22,23]. Cancer stem cells are self-renewing cells that have been suggested to reside within tumors as a source for cancer cells with limited capacity for repeated cell division [20,24]. Our report is the first to show that breast cancer cell survival and metastasis is promoted by DC, and that this effect is obliterated by the FXR antagonist Z-guggulsterone. Our results may pave the way to novel options for breast cancer treatment by blocking DC-induced survival of cancer cells.

## Materials and methods

### Materials

4T1 mouse breast cancer cells were purchased from the American Type Culture Collection (Manassas, VA, USA). RPMI 1640 was from Cellgro (Herndon, VA, USA). Penicillin-streptomycin and Vybrant CM-DiI and Vybrant CM-DiO cell labeling solutions were from Invitrogen (Carlsbad, CA, USA). Fetal bovine serum (FBS) was from Atlanta Biologicals (Lawrenceville, GA, USA). DC and the VEGF receptor (VEGFR)2/Flk-1 antagonist SU5416 was from Sigma Aldrich (St. Louis, MO, USA). Polyclonal anti-β actin goat IgG, monoclonal anti-α tubulin mouse IgG and monoclonal anti-Flk1 mouse IgG were from Santa Cruz Biotechnology, Inc. (Santa Cruz, CA, USA). Polyclonal anti-CD44 rabbit IgG was from Abcam (Cambridge, MA, USA). Cleaved (active) caspase-3 antibody was from Cell Signaling Technology (Beverly, MA, USA). Anti-ceramide rabbit IgG was generated in our laboratory, as described previously [25]. Anti-ceramide mouse IgM (MAS00020) was from Glycobiotech (Kuekels, Germany). Z-guggulsterone was obtained from EMD Biosciences (Gibbstown, NJ, USA). Horseradish peroxidase-conjugated anti-mouse and anti-goat IgG, Cy3-conjugated donkey anti-rabbit IgG, Cy2-conjugated donkey anti-mouse IgG, Cy2-conjugated donkey anti-mouse IgM, μ-chain specific, Cy3-conjugated goat anti-mouse IgG, and normal goat and donkey serum were purchased from Jackson ImmunoResearch (West Grove, PA, USA). The *in situ *terminal dUTP nick-end labeling (TUNEL) fluorescence staining kit was purchased from Oncogene Research Products (San Diego, CA, USA).

### Methods

#### Cultivation and treatment of breast cancer cells

4T1 cells were maintained in RPMI 1640 supplemented with 10% FBS and 1% antibiotic-antimycotic solution at 37°C in a humidified atmosphere containing 5% carbon dioxide. Cells were first cultivated in serum-free medium for 24 hours and then treated with various concentrations of DC or other reagents (for example, Z-guggulsterone, VEGFR-2 antagonist). A stock solution of sodium DC (100 mmol/l) was prepared in water and diluted in the medium to yield the desired final concentrations.

#### Cell labeling

Vybrant CM DiI and DiO are nontoxic, lipophilic, carbocyanine dyes that bind to cellular phospholipid bilayer membranes and are well suited for long-term labeling and tracking of cells. 4T1 cells were labeled with the cell labeling solutions in accordance with the manufacturer's (Molecular Probes, Carlsbad, CA, USA) instructions. Briefly, cells were dissociated by treatment with trypsin and then resuspended at a density of 1 × 10^6 ^cells/ml in serum-free RPMI medium. Cells were then mixed with 5 μl of Vybrant CM-DiI (red) or DiO (green) cell labeling solutions per milliliter of cell suspension and incubated at 37°C for 30 minutes. The labeled suspension was mixed with serum-containing RPMI medium and centrifuged at 1,200 rpm for 5 minutes. The supernatant was removed and the cells gently resuspended in warm medium. The wash procedure was repeated one more time before the cells were plated. After 15 minutes recovery time, labeled cells were observed under an Axiophot fluorescence microscope (Carl Zeiss MicroImaging, Inc., Thornwood, NY, USA) to ensure that all cells took up the dye. Under these conditions we usually observed 100% labeling of cells.

#### *In vivo *analysis of metastasis

**Figure 1:**
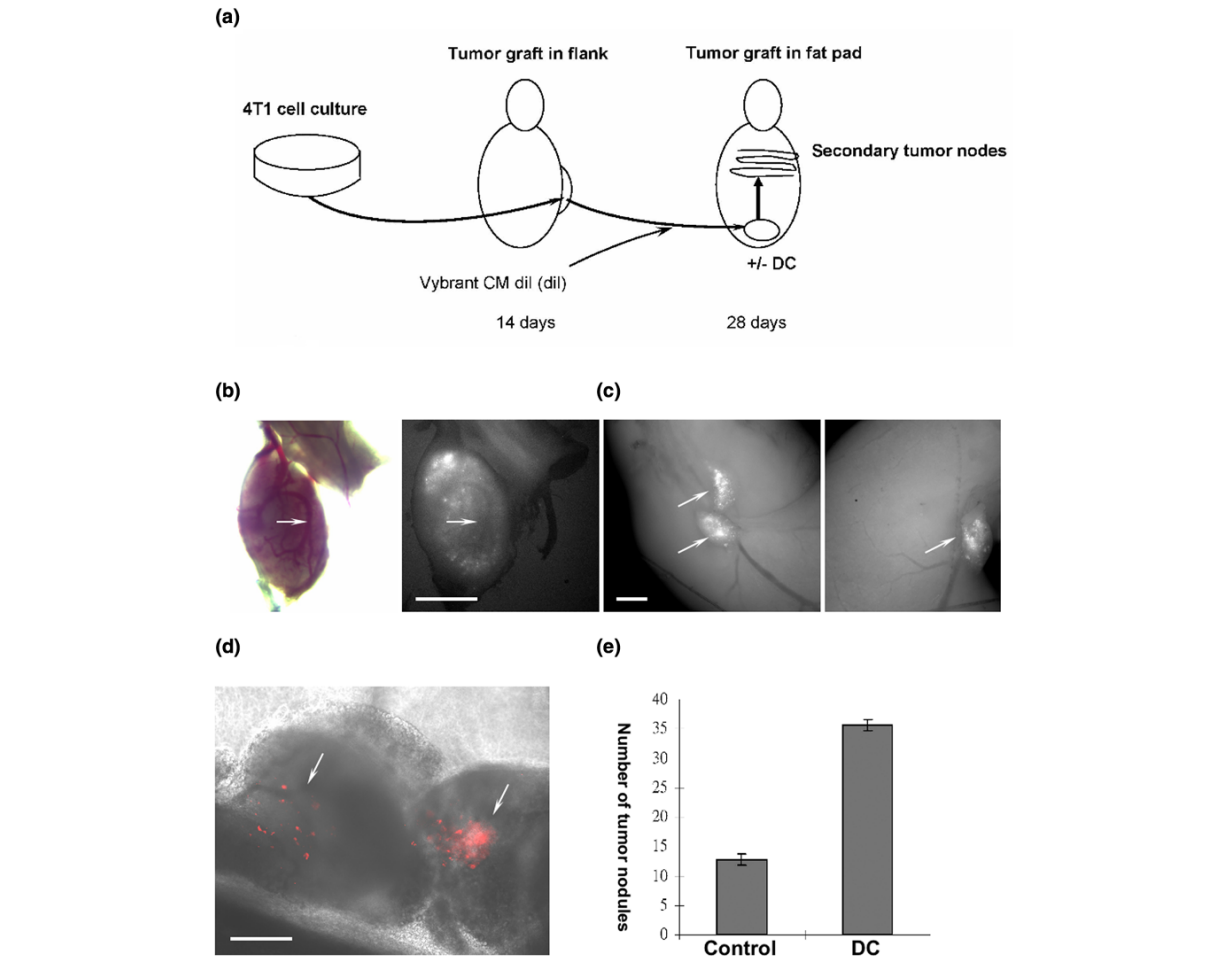
**DC promotes secondary tumor formation from fat pad grafts of 4T1 cells**. **(a) **4T1 tumor and metastasis model. 4T1 cells were injected into the flanks of Balb/c mice. Tumor tissue was extracted from the flanks and chopped into small fragments. Cells were labeled with Vybrant CM-DiI. The labeled tissue fragments were grafted into the fat pads of another series of mice, which were then intraperitoneally injected with vehicle or treated with DC. After 14 days, mice were killed and secondary tumors quantified. **(b) **Brightfield and epifluorescence (Vybrant CM-DiI) image of secondary tumor in lung (arrow points at blood vessel). Scale bar = 1.0 mm. **(c) **Epifluorescence image (Vybrant CM-DiI) in intestine (arrows point at tumor nodules). Scale bar = 1.0 mm. **(d) **Image as in panel c, but overlay with phase contrast image (arrows point at Vybrant CM DiI-labeled cells [red]). Scale bar = 200 μm. **(e) **Mice were treated as described for panel a and the number of secondary tumor nodules in the intestine determined. DC, sodium deoxycholate.

The orthotopic 4T1 mouse breast cancer model in Balb/c mice was used following published procedures [13]. This study was approved by the Institutional Animal Care and Use Committee of the Medical College of Georgia, Augusta, Georgia, USA. The scheme of the *in vivo *model is represented in Figure 1a. Briefly, 10^6 ^4T1 cells were injected into the flanks of Balb/c mice to grow the primary tumor. After 10 to 14 days, tumor tissue was dissected out of the flanks and chopped into small fragments using a surgical blade. Small pieces of approximately 100 cells were labeled with Vybrant CM-DiI (>90% of the cells were labeled), loose cells removed, and the tissue fragments injected into the fat pad (10 pieces/injection) of another Balb/c mouse. Mice (12 in each group) were either injected with vehicle or treated with DC (four intraperitoneal injections of 25 mg/kg per 48 hours starting 24 hours after grafting into fat pad). Two weeks after grafting, mice were killed and the internal organs removed. Metastatic tumor nodules on various organs were counted and analyzed using fluorescence microscopy (to locate Vybrant CM DiI-labeled cells). Vybrant CM-DiI cells within a tumor nodule (0.8 ± 0.2 mm diameter) were quantified by crysectioning five nodules at 10 μm and counting fluorescent cells in consecutive sections throughout the tumor.

#### Hoechst exclusion assay

4T1 cells (living culture) were incubated with 5 μg/ml Hoechst 33342 dye for 30 to 60 minutes at 37°C following a modified protocol previously published [26]. The Hoechst staining was monitored by fluorescence microscopy using the DAPI channel. Micrographs were taken when there was a clear difference in the intensity of the fluorescence signal between slowly and rapidly dividing cells. Slowly dividing cells were identified by staining for Vybrant CM-diI.

#### Immunocytochemistry and flow cytometry

For immunocytochemistry, cells were grown on coverslips and cultivated in the absence or presence of DC or other reagents. Cells were fixed with 4% p-formaldehyde in phosphate-buffered saline (PBS) for 20 minutes and then permeabilized by incubation with 0.2% Triton X-100 in PBS for 5 minutes at room temperature. Nonspecific binding sites were saturated using a blocking solution of 3% ovalbumin/2% donkey serum in PBS. Primary and secondary antibodies were diluted in 0.1% ovalbumin/PBS. Cells were stained with Hoechst to visualize the nuclei. Epifluorescence microscopy was performed with an Axiophot microscope (Carl Zeiss MicroImaging, Inc.) equipped with a Spot II CCD camera. Confocal fluorescence microscopy was performed using a Zeiss LSM510 confocal laser scanning microscope equipped with a two-photon argon laser at 488 nm (Cy2), 543 nm (Cy3), or 633 nm (Cy5, Alexa Fluor 647), respectively.

For flow cytometric analysis, cells were detached using 0.5 mmol/l EDTA/10% FBS and passed through a 40 μm mesh. The cells were resuspended in 100 μl of blocking buffer (3% ovalbumin in PBS) and nonspecific binding sites saturated by incubation for 15 minutes at room temperature. The cells were incubated with primary antibodies (rabbit anti-CD44, 1:50; mouse anti-Flk1, 1:50) and secondary antibodies (Alexa 488 anti-mouse, 1:100; Alexa 647 anti-rabbit, 1:100) diluted in 0.5% ovalbumin/PBS at 4°C for 60 minutes and 45 minutes, respectively. Cells were then washed with PBS, centrifuged, resuspended in 500 μl PBS, and analyzed using a Becton Dickinson FACSCalibur flow cytometer (BD Biosciences, San Jose, CA, USA). Unstained cells were processed similarly, except that in place of primary antibodies they were incubated with 0.5% ovalbumin/PBS. The unstained cells and cells stained with a single combination of primary and secondary antibodies were used to set the gates for the analysis of double stained cells.

#### RT-PCR

Total RNA was prepared from control and DC treated cells using Trizol, following the manufacturer's (Invitrogen) protocol. First strand cDNA was synthesized using Omniscript RT Kit, in accordance with the manufacturer's (Qiagen, Valencia, CA, USA) protocol. PCR was performed for 25 cycles using the following oligonucleotide primers and annealing temperatures: β-actin (sense, 5'-tgacggggtcacccacactgtgcccatcta-3'; antisense, 5'-ctagaagcatttgcggtggacgatggaggg-3'; 56°C) and Flk1 (sense, 5'-taagggcatggagttcttgg-3'; antisense, 5'-cagagcaacacaccgaaaga-3'; 53°C). The amount of template from each sample was adjusted until PCR yielded equal intensities of amplification for β-actin.

#### Western blot analysis

For immunoblot analysis, protein concentrations were determined using the Rc/Dc Protein Assay, in accordance with the manufacturer's (BioRad Laboratories, Inc., Hercules, CA, USA) instructions. Equal amounts of protein were loaded on a 4% to 20% gradient gel and SDS-PAGE was performed using the Laemmli method. For immunoblotting, membranes were first blocked with 5% dry milk in PBST (PBS containing 0.1% Tween-20) and incubated with primary antibodies (anti-Flk1, 1:1,000; anti-active caspase 3, 1:500) diluted in the blocking buffer overnight at 4°C. Membranes were then washed with PBST three times and incubated with the appropriate horseradish peroxidase-conjugated secondary antibodies (1:2,000) for 1 hour at room temperature. Membranes were washed with PBST at room temperature three times (15 minutes each). Bands were detected by using a chemiluminescence system and exposure to X-ray film. Membranes were then stripped and re-probed, as described above, with either anti-β-actin (1:1,000) or anti-α-tubulin (1:1,000) to confirm equal loading.

#### Ceramide analysis by high-performance thin layer chromatography

Total lipids were extracted from cells by sonicating cell pellets in a mixture of chloroform:methanol (2:1; vol/vol). After 1 hour of extraction (on a magnetic stirrer), the solution was centrifuged at 4,000 rpm for 10 minutes. The supernatant was transferred to a clean glass tube, whereas lipids from the pellet were re-extracted in chloroform:methanol (2:1; vol:vol) for 1 hour. The resulting solution is centrifuged again and the supernatant pooled with the supernatant from the first extraction. The pellet is dried down, dissolved in 2% SDS, and used for protein estimation using BCA method (Pierce, Rockford, IL, USA). The supernatant is dried down completely and resuspended in a small volume, based on total protein content of the sample. These samples were resolved by high-performance thin layer chromatography using the running solvent chloroform:methanol:acetic acid (95:4.5:0.5; vol:vol) for the separation of ceramide. Individual bands were visualized by staining with 3% cupric acetate in 8% phosphoric acid and identified by comparing them to the migration distance of standard ceramide [1].

#### Analysis of cell death

For the analysis of cell death, cells were incubated in the absence of serum with or without the addition of DC or other reagents. At the end of the treatment period, cells were detached by trypsinization and pooled with floating cells collected from the medium. Cells were washed with PBS and stained with propidium iodide for 30 minutes at room temperature. Cells were washed with PBS and analyzed by flow cytometry to measure cell death. Results are represented as mean ± standard deviation of four independent experiments.

## Results and discussion

### DC promotes the formation of secondary tumors containing slowly dividing, CD44^+^/Flk-1^+ ^4T1 cells

**Figure 2:**
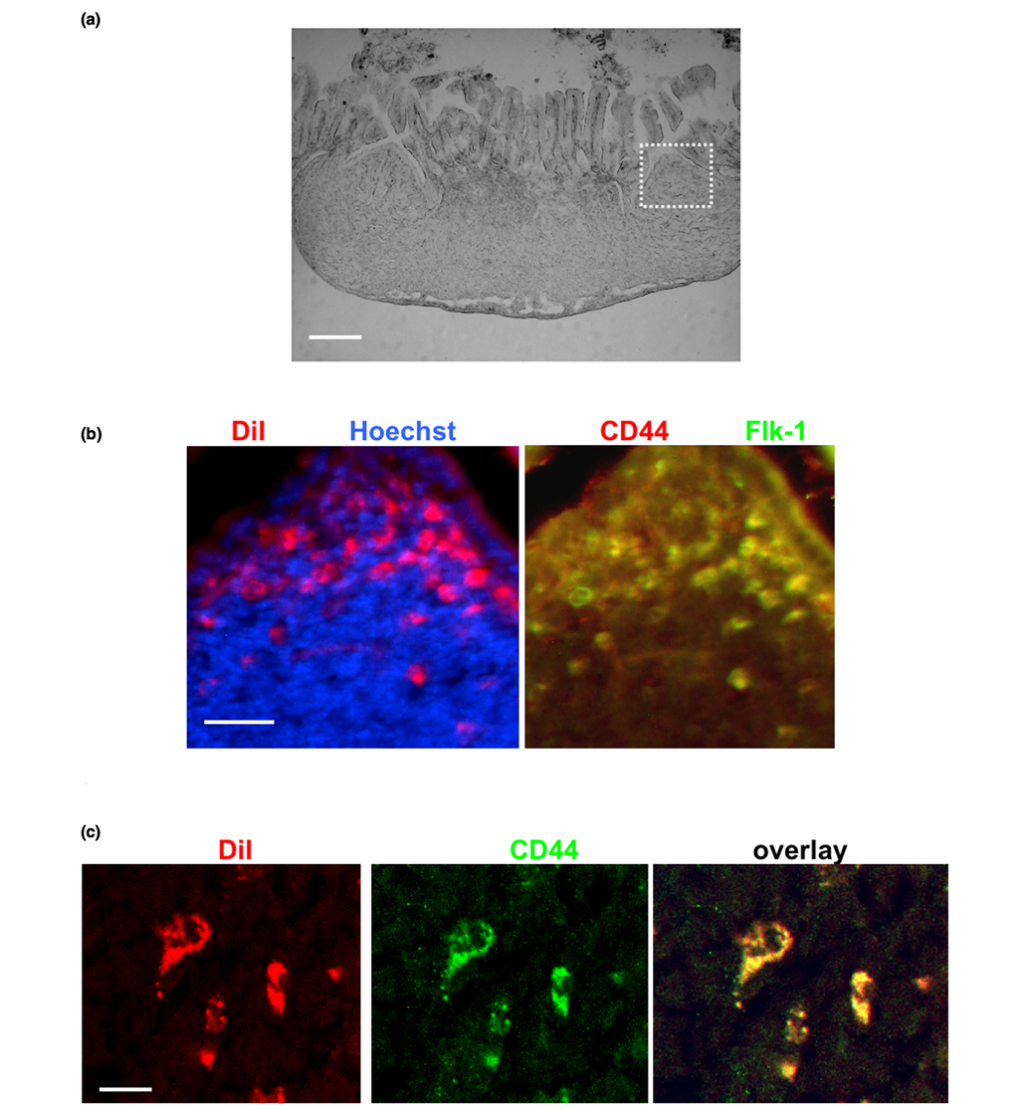
**Secondary tumors contain slowly dividing CD44^+^/Flk-1^+ ^cells**. **(a) **Cryosection of secondary tumor nodule in intestine (differential contrast). The white box shows an area that is further characterized in panel b. Scale bar = 100 μm. **(b) **Cryosection as in panel a used for immunocytochemistry. Note that Vybrant CM DiI-labeled cells (left panel [red]) express CD44 (right panel [red]) and Flk-1 (right panel [green]). Scale bar = 20 μm. **(c) **Another area of the secondary tumor model at higher magnification showing co-distribution of Vybrant-CM DiI and CD44. Preservation of Vybrant CM-DiI staining indicates slow cell division. Scale bar = 5 μm.

Previously, we reported that DC increases cell survival and migration of MDA-MB-231 cells *in vitro *[1]. To test the effect of DC on tumor formation *in vivo*, we used a well established syngeneic and orthotopic 4T1 mouse breast cancer cell model in Balb/c mice [13]. 4T1 cells were injected into the flanks of Balb/c mice and a primary tumor generated (Figure 1a). Primary tumor tissue was extracted from the flanks and chopped into small fragments. Cells in the tissue fragments were labeled with the vital fluorescent dye Vybrant CM-DiI and injected into the fat pads of another series of mice (Figure 1a). Engraftment was confirmed by analyzing tumor nodules in the fat pad. The flank-to-fat pad graft procedure ensured that cells in secondary tumors were truly emigrating from the fat pad graft and were not just diffusing as single cells. DC was administered intraperitoneally at a dose of 25 mg/kg every other day. After secondary tumors had formed, mice were killed and the tumor nodules analyzed using fluorescence microscopy. Secondary tumor nodules were detected in lung (Figure 1b), liver, bone, and intestine (Figures 1c, d and 2a; Figure 2a shows phase contrast and Figure S1A [see Additional data file 1] shows hematoxylin and eosin staining of a cryosection). Figure 1e shows that treatment with DC increased the number of intestinal tumor nodules by threefold, suggesting that DC promoted cell survival, proliferation, or migration of 4T1 cells.

In each primary tumor nodule in the fat pad and in the secondary tumor nodules derived from them, we detected a group of Vybrant CM DiI-labeled cells (0.3 ± 0.1% of total cells in a secondary tumor nodule) surrounded by unlabeled cancer cells that formed the majority (>99% of all cells) of the tumor mass (Figures 1d and 2b). Only labeled cells were able to generate unlabeled progeny cells by repeated cell division, whereas unlabeled cells could not convert into labeled cells. Because each secondary nodule contained Vybrant CM DiI-labeled cells, our results suggest that a portion of labeled cells migrated from the fat pad to the intestine and divided slowly, as may be concluded from the retention of the Vybrant CM-DiI fluorescence. However, these cells also gave rise to rapidly dividing cancer cells, forming the major tumor mass, which lost the fluorescent signal due to dilution of the dye. Alternatively, labeled and unlabeled cells may have migrated together and formed a secondary tumor containing unlabeled cells outgrowing the labeled cells. However, the probability of this event would not be higher than that of single cell migration. Because the secondary tumors contained a portion of labeled cells it is rather likely that these tumors were formed from labeled cells that divided asymmetrically and gave rise to rapidly dividing cells that became unlabeled.

The transition of slowly to rapidly dividing cells has been hypothesized to be characteristic for cancer stem cells [20,24]. The expression of CD44 is another marker that has been used to identify cancer stem cells [21,22]. We cryosectioned the tumor nodules and determined the expression of CD44 using immunocytochemistry. Figure 2b, c shows that the Vybrant CM DiI-stained cells were co-stained for CD44, suggesting that they showed a feature of cancer stem cells [see Figure S1B in Additional data file 1 for individual color channels] [20,21,27]. Because these cells were always located in close vicinity to blood vessels, we determined the expression of Flk-1, a VEGF receptor (VEGFR2) expressed in 4T1 cells [28,29]. Indeed, Vybrant CM-DiI^high^/CD44^+ ^cells were also positive for Flk-1 (Figure 2b). In summary, these results suggested that a subpopulation of slowly dividing cells formed secondary tumor modules after having emigrated from the primary nodules in the fat pad graft. Further, our data indicate that migration or metastasis of these cells was promoted by DC.

### 4T1 cancer cells divide asymmetrically *in vitro*

**Figure 3:**
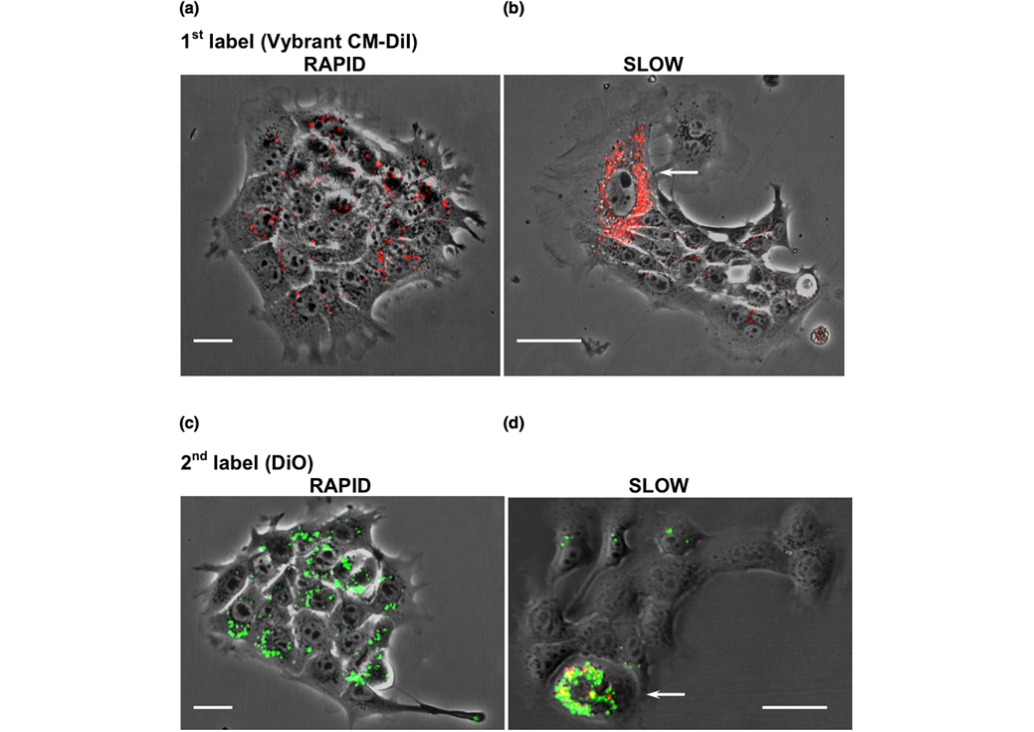
**Slowly dividing cells divide asymmetrically**. **(a, b) **4T1 cells were labeled with Vybrant CM-DiI (red fluorescence) and then single cells were seeded into individual wells of 48-well plates (one cell/well). (Panel a) Forty-six out of 48 wells showed homogenous distribution of Vybrant CM-DiI to the daughter cells (rapidly dividing cells). (Panel b) Two out of 48 wells showed a few highly labeled cells (slowly dividing cells), whereas the rest of the colony became gradually unlabeled (rapidly dividing cells). This heterogeneous distribution of Vybrant CM-DiI suggests asymmetric cell division of slowly dividing cells giving rise to one daughter cell that stays slowly dividing and retains the label (self-renewing cell), whereas the other daughter cell converts into a rapidly dividing cell. **(c, d) **Colonies as shown in panels a and b were dissociated, labeled with DiO (green fluorescence), and again seeded as single cells (one cell/well). (Panel c) Colonies formed from rapidly dividing cells (no red fluorescence) continued to divide rapidly and showed homogenous distribution of DiO until the fluorescence signal was diluted below the threshold of detection. (Panel d) Colonies formed from slowly dividing cells (red fluorescence from Vybrant CM-DiI labeling was still visible) again showed asymmetric cell division giving rise to slowly dividing cells that were labeled with both Vybrant CM-DiI and DiO (arrow), and rapidly dividing cells that became unlabeled. A schematic representation is provided in the additional materials [see Figure S2 in Additional data file 1]. Scale bars = 5 μm.

Retention of Vybrant CM-DiI in a subpopulation of cells within the secondary tumors suggested that slowly dividing cells self-renewed and gave rise to rapidly dividing cells by asymmetric cell division. To test this hypothesis *in vitro*, 4T1 cells were labeled with Vybrant CM-DiI and seeded as single cells in each well of 48-well plates. Figure 3 shows that colonies with two distinct phenotypes were formed: 46 out of 48 colonies showed homogenous distribution of Vybrant CM-DiI staining (Figire 3a) and, eventually, the fluorescence signal was no longer detectable (Figure 3a); and two out of 48 colonies showed retention of Vybrant CM-DiI in a few cells, whereas the fluorescent label was rapidly lost in the rest of the colony (Figure 3b). As depicted in Figure S2 [see Additional data file 1], this distribution of the fluorescent label was attributed to two distinct modes of cell division: single rapidly dividing cells once again gave rise to other rapidly dividing cells; and single slowly dividing cells gave rise to another group of slowly dividing cells and the majority of rapidly dividing cells. The possibility that labeling cells with the vital dye slowed down cell division was ruled out by comparing the growth rates of labeled and unlabeled cells, which was not found to be different (data not shown). Furthermore, Vybrant CM-DiI and its fluorescent analogs (for instance, DiO) have been widely used to label cells and track cell lineage, migration, and tumor growth in several previous studies, without reported toxicity or negative effects on growth rate [30-32].

**Figure 4:**
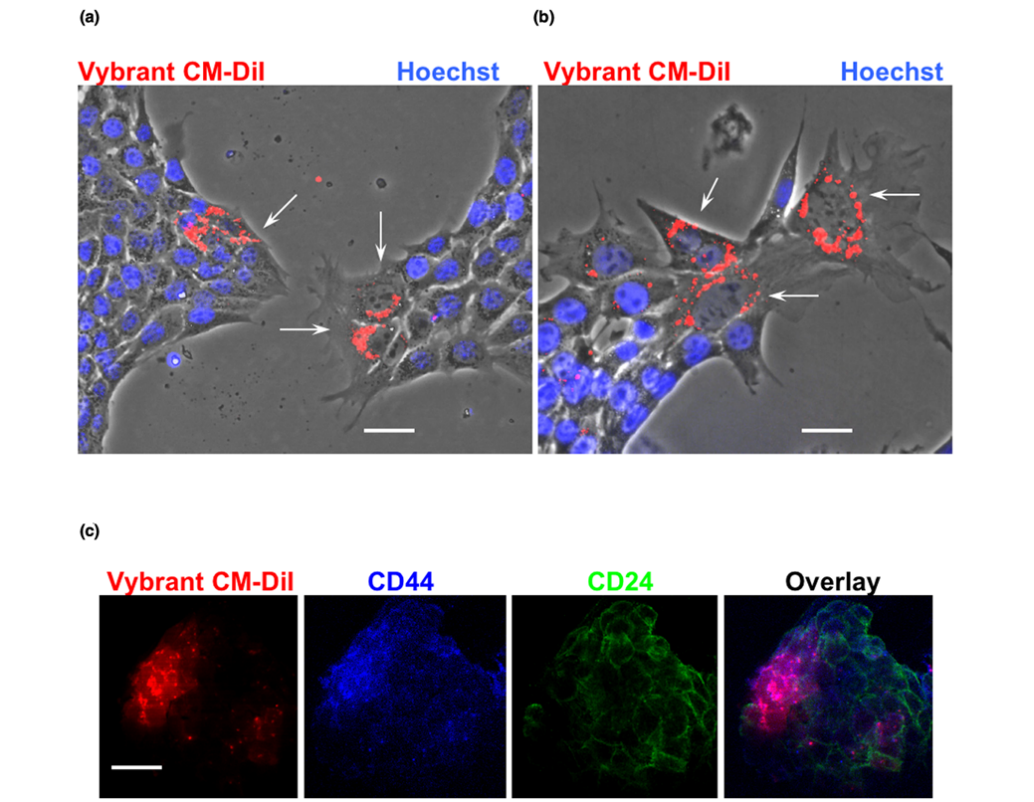
**Slowly dividing CD44^+^/Flk-1^+ ^cells show characteristics of stem-like cancer cells**. **(a) **Hoechst exclusion assay. Vybrant CM DiI-labeled cells were seeded at clonal density and allowed to propagate until reaching 70% confluence. Hoechst exclusion (arrows) was only found in slowly dividing cells that retained Vybrant CM-DiI staining. Scale bar = 5 μm. **(b) **Cells were cultivated as described for panel a and then FACS sorted for high Vybrant CM-DiI staining. After re-cultivation of this cell fraction, Hoechst exclusion (arrows) was again found only in cells that retained Vybrant CM-DiI staining. Scale bar = 5 μm. C. Immunocytochemistry for CD44 and CD24. Note that CD24 shows background expression and that CD44 and Vrybrant CM-DiI are co-distributed (purple in overlay). Scale bar = 20 μm. FACS, fluorescence-activated cell sorting.

The observation that slowly dividing cells gave rise to rapidly dividing cells suggested that the slowly dividing cells divided asymmetrically; one daughter cell remained slowly dividing and therefore retained the fluorescence, whereas the other daughter cell started to divide rapidly and therefore diluted the fluorescent dye over the entire progeny until the label was no longer detectable [see Figure S2 in Additional data file 1]. We then determined whether asymmetric cell division was a stable feature of slowly dividing cells. Alternatively, rapidly dividing cells could become slowly dividing cells and therefore retain fluorescence [see Figure S2 in Additional data file 1]. To test this, we dissociated colonies from the first round of Vybrant CM-DiI labeling, labeled all of the cells with DiO (green fluorescent analog of Vybrant CM-DiI), and again seeded single cells into 48-well plates. The colonies derived from rapidly dividing cells gradually lost green fluorescence, indicating that they continued to divide rapidly (Figure 3c). However, single slowly dividing cells gave rise to rapidly and slowly dividing cells that lost or retained green (DiO) fluorescence, respectively. At the same time, the slowly dividing cells showed residual Vybrant CM-DiI fluorescence from the first round of colony formation (Figure 3d, arrow). This observation was best explained by the assumption that asymmetric cell division was specific for the subpopulation of slowly dividing cells. To test asymmetric cell division further, slowly dividing, Vybrant CM-DiI-labeled cells were separated using fluorescence-activated cell sorting (FACS) and then re-plated at clonal density. Flow cytometry and fluorescence microscopy showed that the progeny distributed into labeled and unlabeled cells at a similar proportion (3 ± 1% retained Vybrant CM-DiI fluorescence) as the cells before FACS (Figure 4a, b). This result suggests that the subpopulation of slowly dividing cells divided asymmetrically and gave rise to self-renewing, slowly dividing daughter cells, while providing the majority of rapidly dividing cancer cells.

To test whether the subpopulation of slowly dividing cells showed other proposed characteristics of cancer stem cells we used two assays: Hoechst exclusion and immunostaining for CD44 and CD24. Some cancer stem cells are known to show less intense staining of nuclear DNA with Hoechst dye because of the expression of the drug resistance transporter (ATP-binding cassette or ABC proteins) [24,33]. Figure 4a shows that cells with an intense fluorescence signal for Vybrant CM-DiI showed none or only a low signal for Hoechst. This result suggested that slowly dividing, DiI-labeled cells exhibited a typical feature of cancer stem cells. Hoechst exclusion was also found with a FACS fraction of highly DiI-labeled cells, but not with unlabeled cells, demonstrating that this feature was typical for the slowly dividing subpopulation of 4T1 cells (Figure 4b).

Slowly dividing, DiI-labeled 4T1 cells were immunostained for CD44, whereas unlabeled cells were not (Figure 4c). Consistent with previous studies [34,35], immunostaining for CD24 was moderate and did not show preference for co-distribution with CD44^+ ^or CD44^- ^cells (Figure 4c). The lack of CD24 in CD44^+ ^cells is one characteristic of human cancer stem cells, but it is not commonly observed in stem-like cancer cells in mouse [27,34,35]. When cultivated at clonal density, DiI^high^/CD44^+ ^cells lost the cell surface expression of CD44 after one or two cell divisions, resulting in progeny cells with increased proliferation rate. This was concurrent with loss of Vybrant CM-DiI staining. Taken together, our results indicated that slowly dividing, Hoechst^-^/DiI^high^/CD44^+ ^cells showed two features expected from breast cancer stem cells (Hoechst exclusion and CD44 expression). In addition, these cells were self-renewing and converted into rapidly dividing Hoechst^+^/DiI^low^/CD44^- ^cancer cells. However, further studies will be required to determine whether the subpopulation of slowly dividing 4T1 cells are genuine cancer stem cells.

### DC elevates the expression of Flk-1 and increases survival of 4T1 cells by reducing ceramide

**Figure 5:**
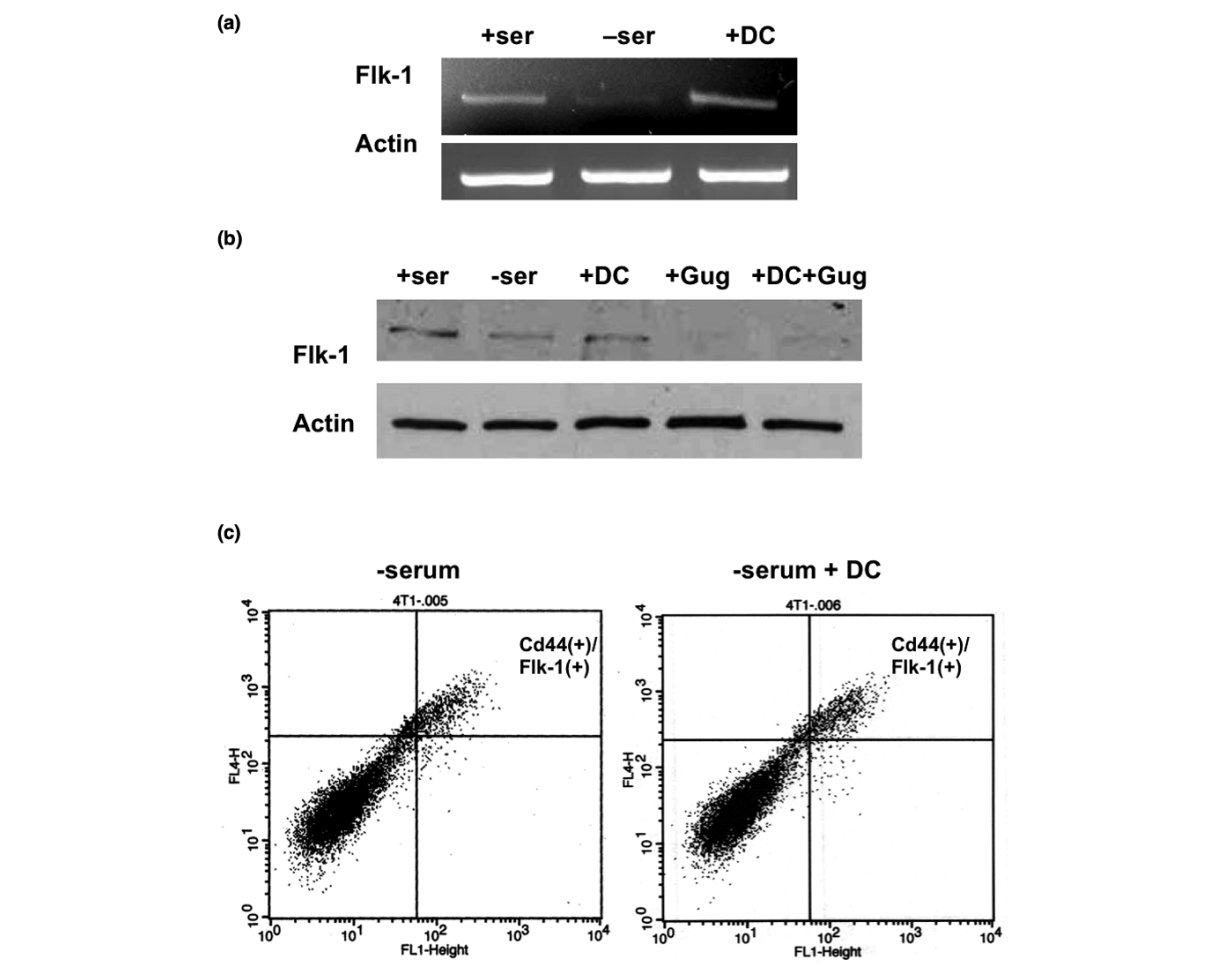
**DC promotes expression of Flk-1, which is prevented by Z-guggulsterone**. **(a) **RT-PCR for the expression of Flk-1 in 4T1 cells. **(b) **Immunoblot for the expression of Flk-1. **(c) **Flow cytometry for the expression of CD44 and Flk-1 in 4T1 cells cultivated in serum-free medium with or without DC. Note that CD44 and Flk-1 were co-expressed. DC, sodium deoxycholate; Gug, guggulsterone (50 μmol/l).

**Figure 6:**
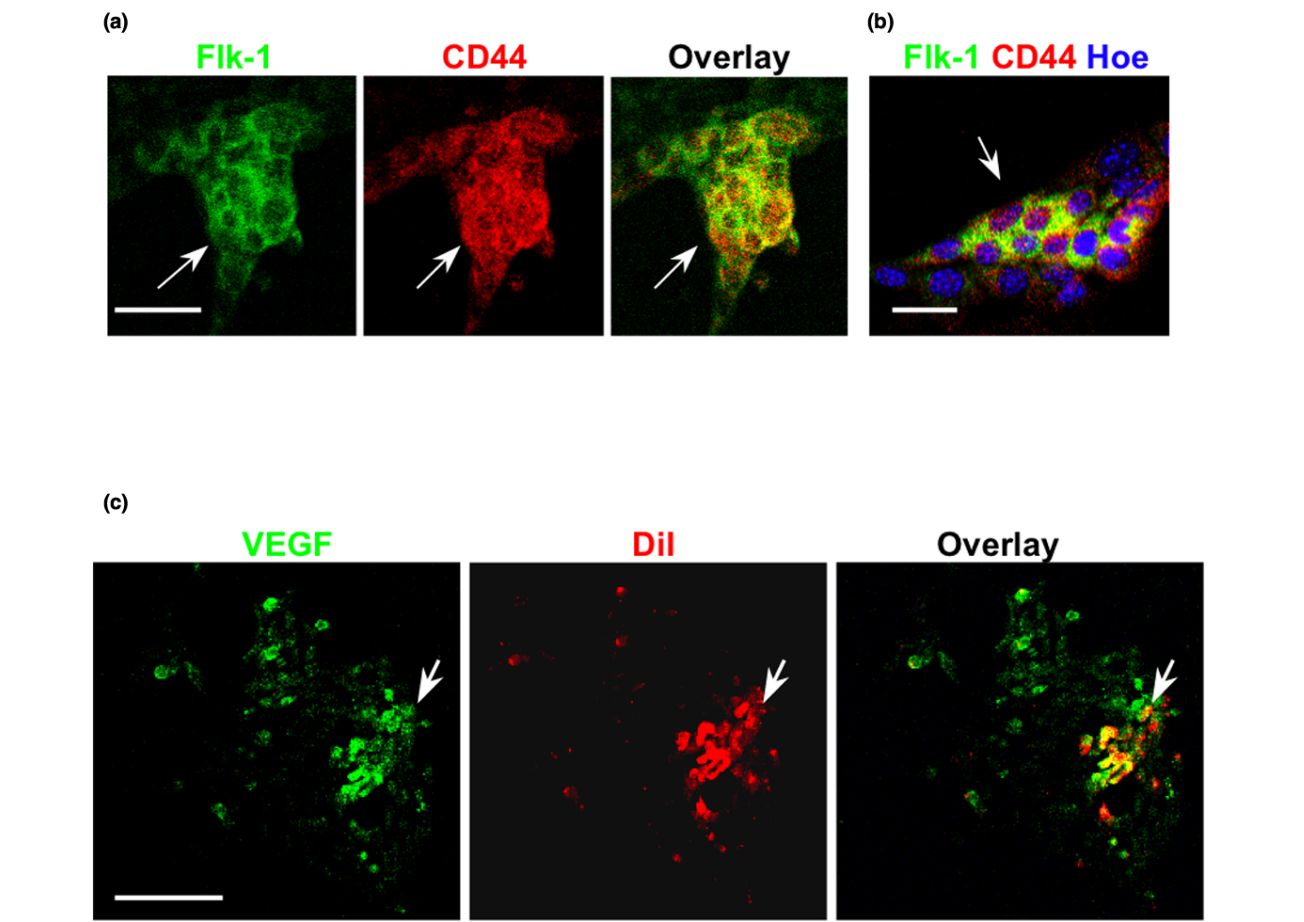
**VEGF and Flk-1 are both expressed in slowly dividing cells**. **(a) **Immunocytochemistry (confocal) for Flk-1 (green) and CD44 (red). Consistent with the results from flow cytometry (Figure 5c), both antigens were co-expressed in slowly dividing cells. Scale bar = 20 μm. **(b) **Immunocytochemistry as in panel a, showing Hoechst staining of nuclei. Scale bar = 5 μm. **(c) **Immunocytochemistry for VEGF (green) indicated that it is also expressed in slowly dividing (Vybrant CM-DiI labeled [red]) cells. Scale bar = 20 μm. VEGF, vascular endothelial growth factor.

In secondary tumors, DiI^+^-labeled cells were found adjacent to newly formed blood vessels (Figure 1b–d). These cells were also stained for CD44 and Flk-1 (Figure 2b, c). Because DC elevated the number of secondary tumors, we tested whether DC enhanced the expression of Flk-1 in 4T1 cells, thereby potentially promoting tumor growth by VEGF [28]. Figure 5a, b shows that incubation of serum-free 4T1 cells with DC increased the mRNA and protein levels of Flk-1. The bile acid receptor (FXR) blocker Z-guggulsterone reduced the DC-induced elevation of Flk-1 protein to less than control levels (Figure 5b). This reduction was consistent with the findings of a recent study [36] that showed that Z-guggulsterone reduces Flk-1 expression and angiogenesis in endothelial cell culture. Flow cytometry and immunocytochemistry showed that Flk-1 and CD44 were co-expressed (Figures 5c and 6a). This finding was consistent with that observed with secondary tumors (Figure 2b), suggesting that Flk-1^+ ^cells showed CD44 expression as one feature found in stem-like cancer cells. CD44^+ ^cells also expressed the Flk-1 ligand VEGF, which is consistent with its autocrine prosurvival effect found in cancer cells (Figure 6b) [37].

**Figure 7:**
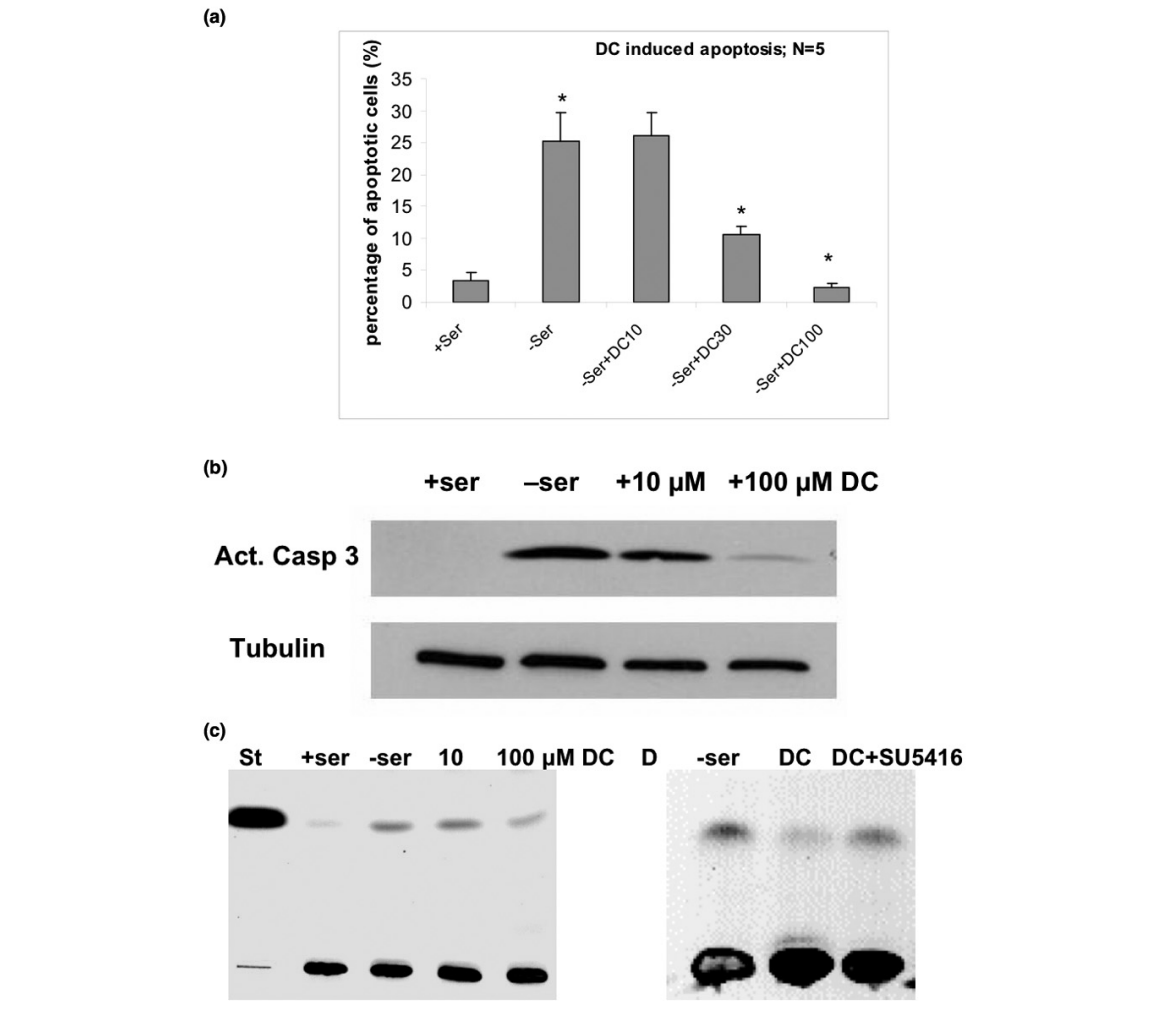
**DC prevents apoptosis by reduction in ceramide; blocking Flk-1 increases ceramide**. **(a) **Apoptosis induced by serum deprivation was quantified by propidium iodide staining and flow cytometry. DC was added to test its dose-dependent effect on the prevention of apoptosis. **(b) **Immunoblot for activated caspase 3. Cells were deprived of serum, lysed, and the protein analyzed by SDS-PAGE/immunoblotting. Note that the addition of 100 μmol/l DC suppressed activation of caspase 3, which was consistent with prevention of apoptosis (see panel a). **(c) **HPTLC analysis of ceramide. Cells were cultivated in the absence of serum and treated with reagents as indicated in the figure. Cellular lipids were then isolated and analyzed by HPTLC. Note that serum deprivation leads to the elevation of ceramide by fourfold. DC suppresses this elevation by 70%. **(d) **HPTLC analysis of ceramide as in panel c. Note that the Flk-1 antagonist SU5416 (10 μmol/l) obliterated DC-induced reduction of ceramide. DC, sodium deoxycholate; HPTLC, high performance thin layer chromatography.

Because DC elevated the expression of the prosurvival factor Flk-1, we tested whether DC reduced cell death via VEGF/Flk-1-dependent cell signaling. Incubation with 30 or 100 μmol/l DC for 48 hours reduced the number of dead (propidium iodide stained) cells by 50% or 80%, respectively (Figure 7a). The Flk-1 antagonist SU5416 completely obliterated the protective effect of DC, suggesting that VEGF and Flk-1 are required for DC-mediated survival of CD44^+ ^cells. Although SU5416 also antagonizes Flt-3 and c-kit, the observations that DC elevated the expression of Flk-1 and that SU5416 obliterated DC-mediated cell survival suggest that SU5416 acted specifically on Flk-1 [31,38]. To identify the mechanism underlying DC-enhanced cell survival, we tested activation of caspase 3 and the level of ceramide, a sphingolipid that induces apoptosis in a variety of cancer cell types [39-42]. Immunoblotting showed that 100 μmol/l DC inhibited activation of caspase 3 induced by serum deprivation (Figure 7b). High-performance thin layer chromatography showed that, when cultivated in serum-free medium, the ceramide level of 4T1 cells increased by fourfold (lane 3, Figure 7c) over serum controls (lane 2, Figure 7c). DC (100 μmol/l) reduced the ceramide level (lane 4) to less than half of that found with serum-free cells. This effect was obliterated by SU5416 (Figure 7d), which is consistent with the observation that SU5416 reversed DC-induced cell survival.

**Figure 8:**
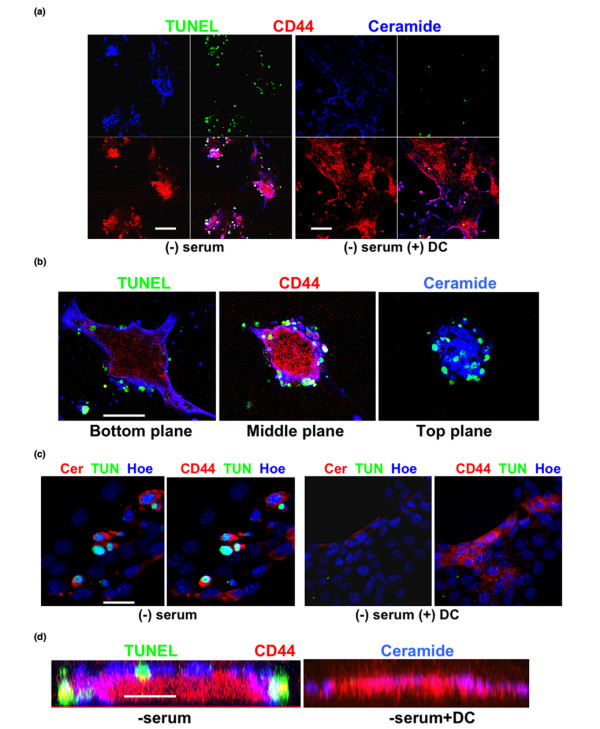
**Ceramide is elevated in CD44^+ ^cells, which is suppressed by DC**. **(a) **4T1 cells were cultivated in serum-free medium and immunocytochemistry was performed for ceramide (blue) and CD44 (red). Apoptotic cells were identified by TUNEL staining (green). Note that CD44^+ ^cells contained high levels of ceramide, which induced apoptosis in the periphery of CD44^+ ^cell clusters (left panel). Ceramide elevation and apoptosis was prevented by DC (right panel). Scale bar = 100 μm. **(b) **Immunocytochemistry as in panel a (without DC), showing three optical planes as obtained from confocal laser scanning immunofluorescence microsocopy (Z-scan). Note that CD44^+^/ceramide^+^/TUNEL^+ ^cells are located at the periphery of CD44^+ ^cell clusters. Scale bar = 20 μm. **(c) **Immunocytochemistry as in panel a, showing Hoechst staining of nuclei. Note that the majority of the CD44^+^/ceramide^+ ^cells were apoptotic (TUNEL^+^), unless cells were incubated with DC (panels on the right). Scale bar = 10 μm. **(d) **Confocal scanning as described in panel b, but reconstruction of the fluorescent signal distribution in a Z-scan (z-axis is shown). Note that the peripheral distribution of ceramide^+^/TUNEL^+ ^cells is consistent with the results shown in panel b. Scale bar = 10 μm. DC, sodium deoxycholate; TUNEL, terminal dUTP nick-end labeling.

We then used two anti-ceramide antibodies (rabbit IgG generated in our laboratory and mouse IgM MAS00020) to detect ceramide-containing 4T1 cells using immmunocytochemistry. Figure 8a–d shows that CD44^+ ^cells, in particular in the periphery of CD44^+ ^cell clusters, expressed high levels of ceramide and underwent apoptosis after serum deprivation, as determined by TUNEL assays (white staining in overlay; Figure 8b, middle panel). Incubation with 100 μmol/l DC prevented apoptosis of these cells and resulted in expansion of rapidly dividing CD44^- ^cells surrounding the CD44^+ ^cell clusters. Apoptosis was restored when cells were incubated with Z-guggulsterone or C2-ceramide (not shown). In summary, our results suggest that DC-induced elevation of (activated) Flk-1 leads to the reduction in pro-apoptotic ceramide, which promotes survival of CD44^+ ^breast cancer cells and rapid expansion of their CD44^- ^progeny cells.

## Conclusion

In our previous studies we showed that the secondary bile acid DC induces migration and promotes survival of human breast cancer MDA-MB-231 cells [1]. To determine the effect of DC on tumor growth *in vivo*, we used murine 4T1 cells in a syngeneic mouse model for breast cancer metastasis. The 4T1 model does not need immunocompromised mouse hosts and shows rapid metastasis to all inner organs and bone, which is not completely achievable with MDA-MB-231 cells. Using this model, we found that DC increases the numbers of metastatic nodes, clearly showing that DC promotes tumorigenesis and/or metastasis from 4T1 cells.

This result is consistent with data obtained *in vitro *showing that DC protects 4T1 cells from apoptosis induced by serum deprivation. The protective concentration is higher than that found with MDA-MB-231 cells, which may be due to cell-specific differences between human and mouse breast cancer cells. This concentration is within the range of that found for bile acid elevation in breast cyst fluid, which has been suggested to increase the risk for breast cancer [2]. Most intriguingly, our results demonstrate that bile acids, in particular DC, are not *a priori *pro-apoptotic, but they may very well support survival and metastasis of breast cancer cells *in vitro *and *in vivo*.

Several lines of evidence suggest that *in vivo*, secondary tumors are initiated by a specific subpopulation of slowly dividing, self-renewing cancer cells. First, each of the intestinal tumor nodules contains Vybrant CM DiI-labeled cells that can only originate from labeled cells having emigrated from the fat pad graft. Because these cells retain staining, but at the same time are surrounded by nonlabeled tumor cells derived from them, they have divided asymmetrically. Second, the slowly dividing, Vybrant CM DiI-labeled cells are positive for CD44, which has been proposed to be a stem cell marker. Finally, it is known from a variety of stem or progenitor cell types that VEGF isoforms and their receptors are co-expressed with CD44 and that VEGF is an essential autocrine and anti-apoptotic factor in 4T1 cells [37,43-46]. These observations prompted us to determine DC-induced survival of a subpopulation of CD44^+ ^cells in a 4T1 cell culture.

It has been reported for endothelial cells that inhibition of Flk-1 induces elevation of ceramide, leading to apoptosis by activation of caspase 3 [47,48]. Because VEGF and Flk-1 are co-expressed in 4T1 cells, we tested whether the anti-apoptotic effect of DC involves elevation of Flk-1 and reduction in ceramide and active caspase 3. Flk-1 is specifically expressed in CD44^+ ^cells, suggesting that stem-like cancer cells are protected by DC-induced elevation of Flk-1. However, when counting CD44^+^/Flk-1^+ ^cells we found that the proportion of these cells was not increased. Therefore, we conclude that DC increases the expression level of Flk-1 in CD44^+ ^cells without increasing the number of these cells. Alternatively, the number of CD44^+^/Flk-1^+ ^cells is initially increased, but then these cells rapidly convert into CD44^-^/Flk-1^- ^cancer cells.

**Figure 9:**
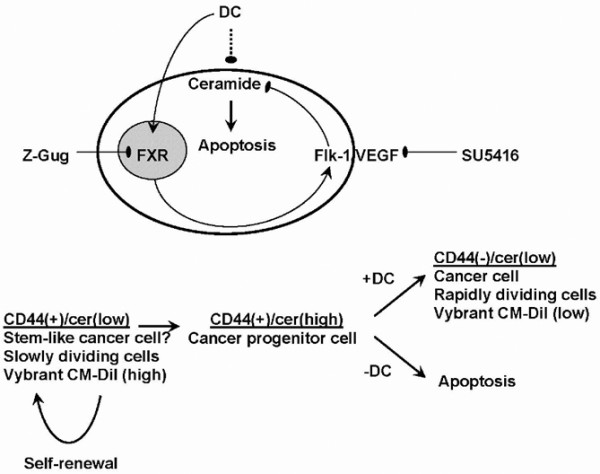
**Model for DC-sustained cell survival by reduction in ceramide-induced apoptosis in BCPCs**. Initially, CD44^+ ^stem-like cancer cells are low in ceramide and show asymmetric cell division, giving rise to one stem-like cancer cell and one breast cancer progenitor cell (BCPC). Serum deprivation (growth factor withdrawal) and loss of VEGF receptor (VEGFR) activation leads to increased ceramide levels and the induction of apoptosis in BCPCs. DC induces VEGFR expression, which prevents ceramide elevation and apoptosis in BCPCs, and promotes metastasis and growth of secondary tumors. Apoptosis is restored by blocking the DC receptor FXR with Z-guggulsterone (Z-Gug) or inhibiting VEGFR with SU5416. BCPC, breast cancer progenitor cell; Cer, ceramide; DC, sodium deoxycholate; FXR, farnesoid X receptor; VEGFR, vascular endothelial growth factor receptor;

Figure 9 shows a model that delineates how DC-induced reduction of ceramide will lead to survival of peripheral CD44^+ ^cells, which is then followed by rapid conversion into CD44^- ^cells. In the case that ceramide is elevated in the peripheral cells, they undergo apoptosis by caspase 3 activation. Only self-renewing (stem-like?) cancer cells in the center of the CD44^+ ^cell clusters persist. However, if ceramide is reduced, then peripheral cells may divide asymmetrically giving rise to self-renewing CD44^+ ^and rapidly dividing CD44^- ^cancer cells. Most intriguingly, we have found a similar mechanism for ceramide-induced apoptosis in asymmetrically dividing, embryonic stem cell derived cells that undergo apoptosis versus differentiation to neural progenitor cells [49]. These cells divide asymmetrically giving rise to one daughter cell that dies from ceramide-induced apoptosis, whereas the other one survives, proliferates, and then terminally differentiates. It is quite possible that the surviving neural progenitor cells behave similarly to the breast tumor cells or breast cancer progenitor cells (BCPCs) in that prevention of ceramide-induced apoptosis promotes survival and proliferation of the progenitor cells. Unlike neural progenitor cells, however, BCPCs do not become postmitotic and differentiate in a controlled way, but continue to divide for numerous cell cycles and grow a tumor mass.

This is the first report describing that DC reduces ceramide-induced apoptosis in breast cancer cells. With respect to tumor stem cells, we propose that ceramide elevation may eliminate BCPCs, which is blocked by DC. Our results suggest that VEGF/Flk-1 is a key factor in DC-induced reduction in ceramide. The effect of Z-guggulsterone on the protein level of Flk-1 and the Flk-1 antagonist SU5416 on ceramide and apoptosis suggests that DC promotes cell survival via a novel FXR-to-Flk-1 cell signaling pathway reducing pro-apoptotic ceramide in BCPCs.

Our study also suggests that administration of Z-guggulsterone and reducing serum bile acid levels could be useful in lowering the risk for metastasis. This hypothesis is consistent with recent studies reporting that Z-guggulsterone induces apoptosis in breast cancer cells and that probiotic administration of lactobacillus reduces serum DC and breast tumor growth in mice [50-54]. Although the direct causal link between the reduction of DC levels and tumor growth has not yet been demonstrated in these models, these results are consistent with epidemiological studies showing that the risk of breast cancer is lower in women who have consumed lactobacillus-containing food (for example, yoghurt and miso soup with living cultures) [50,55]. Our study could provide a mechanistic explanation in that lowering DC serum levels (for instance, by probiotic therapy) or blocking FXR with Z-guggulsterone would increase ceramide-induced apoptosis in BCPCs and reduce growth or spread of the tumor. Alternatively, ceramide could be directly administered to BCPCs or elevated by treatment with VEGFR2/Flk-1 antagonists. In future studies we will further investigate the prosurvival cell signaling pathway induced by DC and test the therapeutic potential of serum DC reduction in breast cancer.

## Abbreviations

BCPC: breast cancer progenitor cell; DC: sodium deoxycholate; FACS: fluorescence-activated cell sorting; FBS: fetal bovine serum; FXR: farnesoid X receptor; PBS: phosphate-buffered saline; PBST: phosphate-buffered saline containing 0.1% Tween-20; RT-PCR: reverse transcription polymerase chain reaction; TUNEL: terminal dUTP nick-end labeling; uPA: urokinase like plasminogen activator; VEGF: vascular endothelial growth factor; VEGFR: vascular endothelial growth factor receptor.

## Competing interests

The authors declare that they have no competing interests.

## Authors' contributions

KK performed cell culture experiments and part of the animal studies, the lipid and RT-PCR analysis, analyzed data, and wrote the initial drafts of the manuscript. GW performed immunocytochemistry, part of the animal studies, and analyzed data. DR assisted in performing the RT-PCR and immunoblot analyses. EB designed the study, and edited and wrote the final manuscript.

## Supplementary Material

Additional file 1 Figures S1 and S2. Figure S1A shows hematoxylin and eosin (H&E) staining of a cryosection of a secondary tumor nodule. Figure S1B shows the single color channels of the tumor section shown in Figure 2B and 2C, stained for CD44, Flk1, Hoechst, and Vybrant CM DiI. Figure S2 shows a schematic representation of the anticipated results of the experiment described in Figure 3. It depicts the fate of labeled cells following the two alternative modes of cell division Click here for file
