# Supplementary material for: Deoxycholate promotes survival of breast cancer cells by reducing the level of pro-apoptotic ceramide
Source: Breast Cancer Res. 2008 Dec 16;10(6):R106. doi: 10.1186/bcr2211 (PMC2656903; doi:10.1186/bcr2211)

Supplemental Figure 1

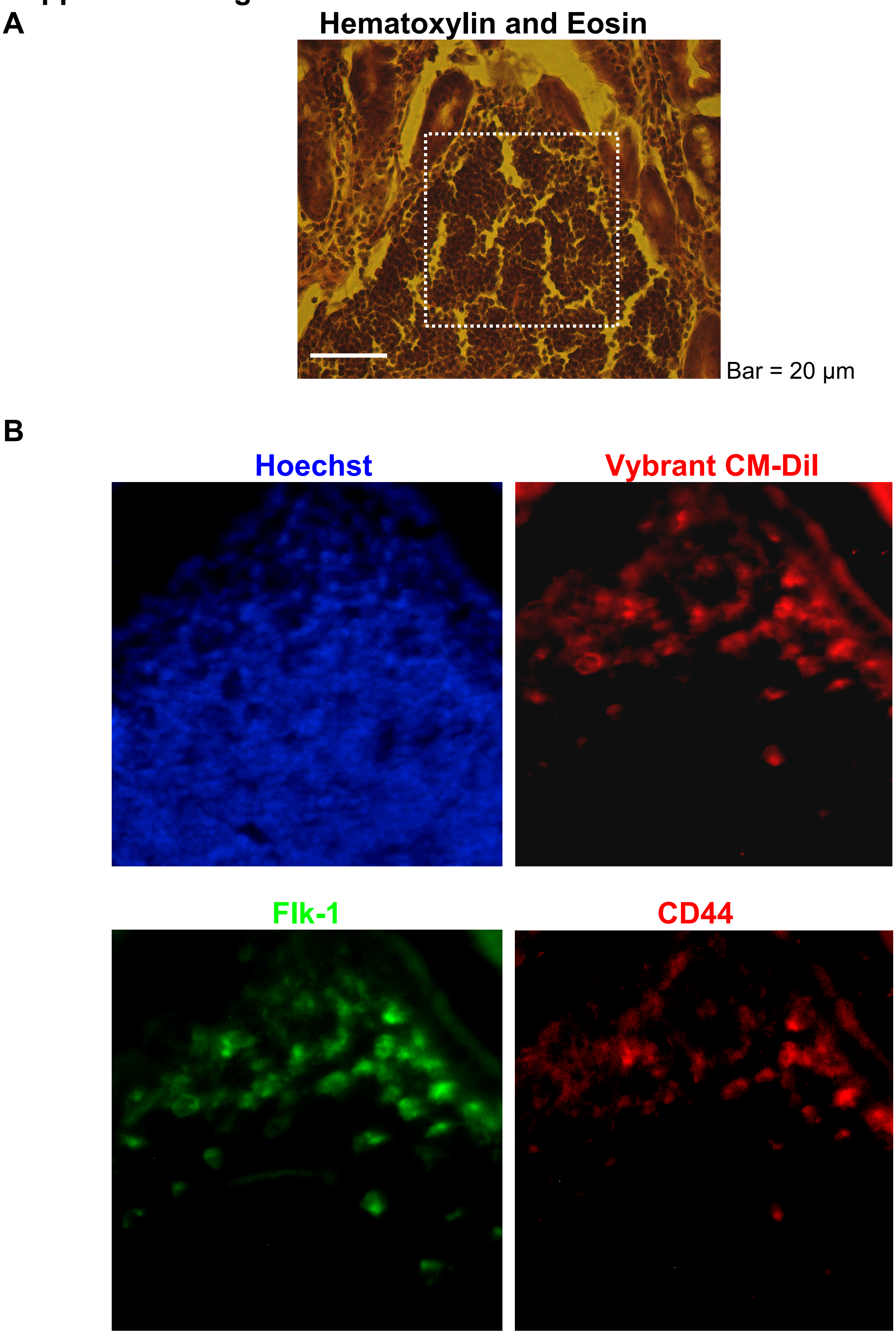

Supplemental Figure 2

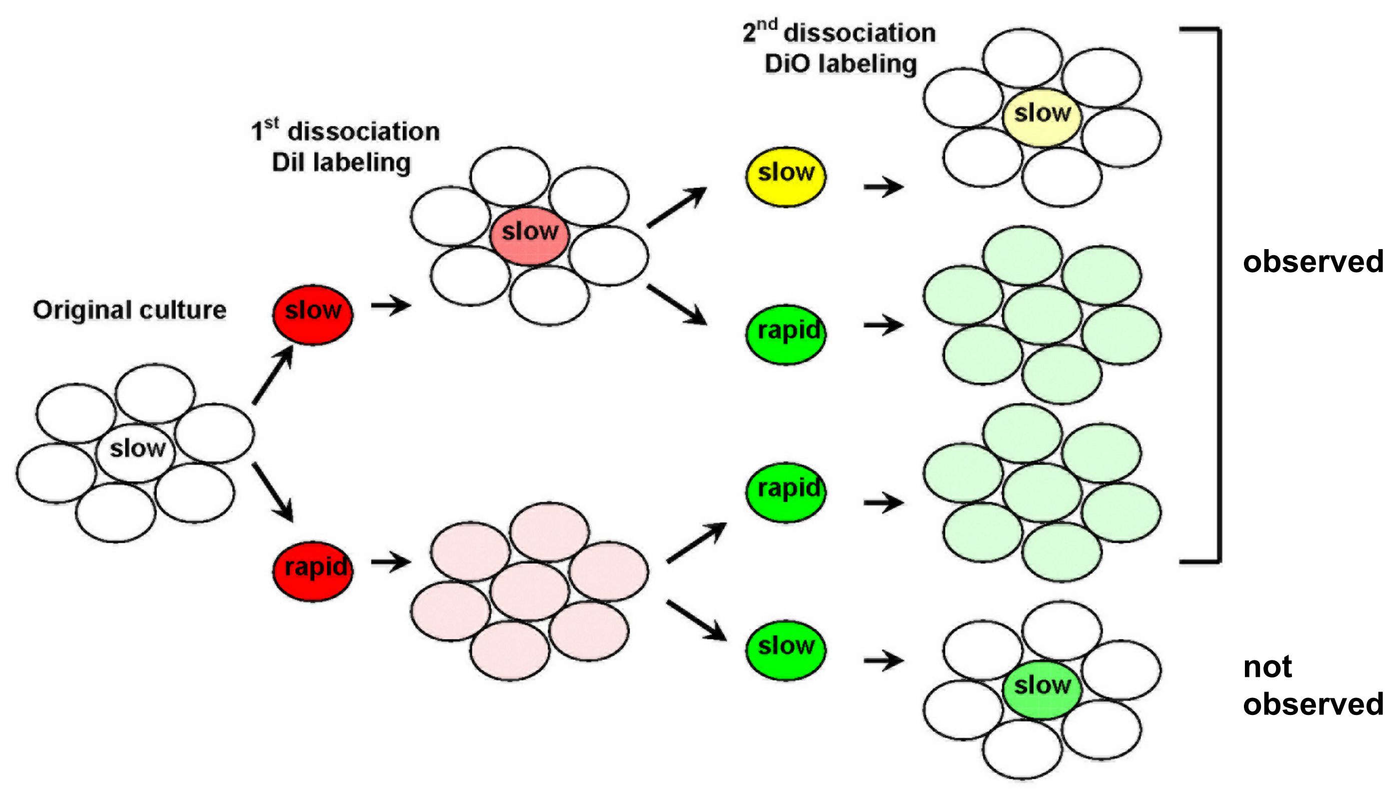

Supplement: Additional file 1 — Figures S1 and S2. Figure S1A shows hematoxylin and eosin (H&E) staining of a cryosection of a secondary tumor nodule. Figure S1B shows the single color channels of the tumor section shown in Figure 2B and 2C, stained for CD44, Flk1, Hoechst, and Vybrant CM DiI. Figure S2 shows a schematic representation of the anticipated results of the experiment described in Figure 3. It depicts the fate of labeled cells following the two alternative modes of cell division [file bcr2211-S1.pdf]
